# Minimising the stress of weaning of beef calves: a review

**DOI:** 10.1186/1751-0147-53-28

**Published:** 2011-05-13

**Authors:** Daniel Enríquez, Maria J Hötzel, Rodolfo Ungerfeld

**Affiliations:** 1 Laboratório de Etologia Aplicada e Bem-Estar Animal, Departamento de Zootecnia e Desenvolvimento Rural, Universidade Federal de Santa Catarina, Florianópolis, SC, Brazil; 2 Departamento de Fisiología, Facultad de Veterinaria, Montevideo, Uruguay

**Keywords:** mother-calf bond, separation, distress, weaning practices, behaviour, animal welfare

## Abstract

Weaning of beef calves is usually done abruptly and early compared to the natural weaning of the species, and is associated with simultaneous exposure of calves to a range of social and environmental stressors. Behavioural and physiological responses to weaning indicate detrimental effects on the welfare of these animals. The development and assessment of weaning methods aiming at reducing or avoiding this problem must be supported by scientific knowledge of the morphological, physiological and psychological mechanisms involved in the establishment, maintenance and braking of the cow-calf bond. Solutions also depend on the understanding of the various stressors associated with weaning, among which are the change in diet, cessation of nursing, separation from the dam, the change to a new spatial environment and the need for social reorganization following removal of the adults from the group. This review discusses these issues and assesses the effectiveness of the methods so far proposed for improving the welfare of beef calves during the weaning period.

## Introduction

In extensive systems, beef calves are usually weaned at around six months of age. The main objective is to improve the cow's body condition, thus preparing her for the forthcoming lactation. Although increasing the reproductive and productive performance of the herd, abrupt weaning is a source of stress for the cow [1-4] and calf [5-8]. This effect is especially acute and prolonged for the calf, which at weaning is subjected to multiple stressors such as the loss of the mother and access to the udder and milk, and changes in the social and physical environment [9,10].

Some management strategies have been proposed that aim to reduce the stress associated with weaning, so that production goals are in line with ethical requirements of society regarding livestock production [11,12]. In general, these techniques involve separating the termination of suckling from the social separation of the calf-dam pair, for example by keeping the calves separated from the dams through fenceline contact for a period before the final separation [5] or with the aid of nose-flaps that allow social contact but not suckling [1].

This paper presents the current knowledge available in the literature on the establishment and maintenance of maternal-filial bond in beef cattle. This bases a discussion on the possible relationship between the mechanisms involved in these processes and the behavioural and physiological response that follows when this bond is severed. Finally, we discuss the advantages and limitations of the methods proposed to improve welfare of calves at weaning. Welfare in this text is understood as the possibility to express natural behaviours and the absence of both suffering and negative influences on the organism which may impair homeostasis.

### Establishment and maintenance of mother-young bond

The dam improves her reproductive success by investing in the survival of the newborn, which she achieves by providing it with care and nutrition [13-15]. A number of morphological, physiological and psychological mechanisms that result in specific behaviours ensure that the young seeks its mother's care and that she will respond accordingly. Under natural conditions, the survival of the newborn depends on the establishment of a strong and lasting social bond with the dam.

The mother-young bond has been defined as a preferential mutual, emotional attachment, of relatively long duration, and that resists temporary separations [16]. It is characterized by affiliative behaviours such as licking, provision of food, warmth and protection, rest in company, synchronization of activities and maintenance of closeness and body care [9,17]. The willingness to establish such an intimate social relationship begins before birth and is reinforced by the contact between mother and young during the first hours after birth [18]. In the dam, this phenomenon involves several physical and physiological changes that occur during pregnancy, parturition and the first contacts with the offspring. The secretion of reproductive hormones such as oestradiol, prolactin and oxytocin and their relative concentrations in blood are of vital importance for the establishment of maternal behaviour in the parturient animal [19]. Previous experience of the dam can also modify the bonding process in some species [19,20]. In cattle, which are characteristically gregarious, the moments before birth are among the few in which the cow seeks to depart from the herd. Although domestic cows seem to adapt their calving strategy to ecological factors ([21]),cattle are usually regarded as hiders. It has been shown that cows isolate themselves from the rest of the herd to calve; this relative isolation allows the association with the newborn during the period of highest sensitivity and predisposition for the establishment of a mother-young bond [22]. This behaviour also increases the efficiency of recognition, reducing the chances of other cows in the group to interfere with the bonding process [23,24].

After birth, the cow licks its calf to clean it, which also stimulates the onset of respiration, circulation and expulsion of the first excretions. In addition to activating these physiological reactions, licking also improves the recognition of the young by the cow [18,25]. The cow sniffs and licks the calf especially during the first two hours after birth, and with decreasing frequency during the following four days [25,26]. The motivation to consume amniotic fluid by the cow can be observed even before birth [27]. Finally, though highly prevalent in the species, the intensity and ability to express maternal behaviour has been related to cow's experience or parity [25,28].

The calf's recognition and attraction to its mother begins within minutes after birth. Such recognition, which is characterized by being exclusive and not transferable, in mammals is most often referred as bonding [21]. After birth, the newborn initiates teat-seeking advances, licking and sucking any protuberance from the dam until it finds the udder [29,30]; the time taken until the calf finds the teat depends on calf vigour, the cow's parity and, most significantly, udder conformation [29-31]. The survival of the newborn depends on it being able to suckle as soon and as efficiently as possible [18,32,33]. Suckling is also essential for the establishment of the bond by the young, and is guaranteed by the innate high motivation of the calf to suck [33,34]. Although in sheep it has been determined that colostrum ingestion appears to be implicated in the establishment of an exclusive preference for the mother [35,36], similar effects have not been described in cattle.

The recognition between mother and young involves all the senses, although for the cow visual recognition appears to be the least important [18,24,32]. Calves can recognize their dam simply through the sounds she emits [37], and can learn to recognize her within 24 h of contact with the dam after birth [38]. The frequency and duration of vocalizations emitted by calves have individual characteristics that favour communication between the pair [39]. However, calves are not highly vocal when the cow is present [21,40], possibly as a strategy to avoid attracting predators, which explains why the cow is less able to recognize its calls as soon as the calf [38].

Contact between the pair during the first hours of life is enough for the establishment of a mother-young bond that grows in strength over time. The bond is stronger in beef than dairy breed [41]. It is maintained and reinforced by the high constancy and frequency of suckling events, which does not seem to change significantly between birth and weaning [17,42], and by the close association and the physical contact that the young keeps with its mother [18]. The bond is mediated in both the dam and the young by the release of hormones and neurotransmitters [10].

Once established, the mother-young bond lasts for several months. For example, in eight-month-old heifers separated for three weeks from their dams, the pattern of social interactions with the dam was minimally affected after reunion, even though nursing had ceased [43]. In another study, three weeks after separation eight-months-old calves showed a preference for their dam compared to other familiar cows [44].

Several neurochemical systems are involved in the process of recognition between the dam and her young, and in the maintenance of the bond. However, as information about these mechanisms in cattle is scarce, data from other species are commonly extrapolated. In mammals there is evidence of direct and indirect participation of hormones and neurochemicals, such as estradiol, oxytocin, prolactin, vasopressin, and several endogenous opioids in the initiation and maintenance of the mother-young bond [45]. Thus the secretion by the cow of hormones such as oxytocin, prolactin [46] and endogenous opioids [47] during lactation could be associated with the strength of the bond. Oxytocin, a hormone that is involved in social attachment in many mammal species [48] and in the stress response of separation from the dam in rat pups [49,50], is released in response to sucking in calves [51]. Cholecystokinin is released in calves in response to both nutritive and non-nutritive sucking [51,52], and has been shown to mediate the vocalization response in rat pups deprived of milk [53]. Besides the loss of milk, the sudden decline in plasma concentrations of hormones and neurotransmitters secreted in association with suckling and milk intake seems to mediate the behavioural responses to abrupt weaning in calves [10]. However, although understanding the natural weaning process would be of interest to improve weaning practices, little is known about the physiological and behavioural process during the waning of the bond to the dam that leads to cessation of suckling [9,19] and most knowledge comes from other species.

### Breaking the mother-young bond: natural weaning and mother-offspring conflict

Under natural conditions, weaning involves the gradual decrease in milk supply from the mother, and a concomitant increase in the intake of solid food by the young, which is accompanied by a gradual reduction in maternal-filial bond [54]. The relationship between the mother and her offspring in mammals can be schematically divided into three stages: during the first months of life the dam seeks and initiates contact with the young; later, the young will be responsible for most nursing and social contact and, finally, the dam starts rejecting some of the nursing attempts until it is permanently stopped [14]. In cattle, the weaning process may be accompanied by an increased aggressiveness of the mother towards her young [18].

In several species, such as sheep, horses, pigs and bison, there is a gradual and slow reduction in maternal care and suckling during natural weaning; for example, the dam produces less milk, initiates fewer sucklings and terminates more, and makes suckling more costly to the young, which forces it to search for other food [21]. The beginning of natural weaning process appears to be related to the age and size of the young. It has been proposed that in most species of ungulates that have not undergone a recent genetic selection weaning occurs after the offspring reaches a weight equivalent to four times the weight at birth [55]. According to some authors, weaning starts when milk is not enough to supply more than 40 to 50% of energy requirements of the young. As the offspring matures and becomes able to obtain food by itself, the time and energy invested by the dam results in proportionately less benefits to the offspring, even though for her it implies an increasing biological cost. Therefore, the weaning process begins when the energy invested by the dam in the care of her offspring is greater than the benefit that this investment represents for the young and, moreover, this investment may put at risk the future reproductive success of the dam. From this point there is a gradual reduction in milk production and maternal care, which allows more energy for a new reproductive cycle; in the process, she starts avoiding the requests for care by the young. In contrast, due to the high benefit the young obtains from milk, it will try to extend lactation and maternal care for as long as possible. This process is known as the maternal-filial conflict [14].

### Weaning in domestic cattle

The most recognized study describing natural weaning in domestic cattle [42], reports that this occurs between 7 and 14 months after birth, with great individual variation. Possibly due to the gradual nature of the changes in diet and time spent close to the mother, no clear behavioural changes are observed during natural weaning. In that study it was reported that even after the cessation of nursing events, calves still kept some closeness to their dams for several months.

In contrast, weaning of calves in conventional systems is usually abrupt and early compared to the natural process. As a consequence, the separation from the dam occurs without the completion of the period of learning and physiological adaptation to the new diet and group composition. In general it is carried out between five and eight months of age, when the lactation peak is over and calves already graze and ruminate, i.e. at a moment when the weaning process is already in progress. However, as suckling and poor nutrition are amongst the most important factors influencing calving interval in beef cattle [56] earlier weaning, usually at about 70-90 days of lactation, is also being recommended to increase reproductive rates in extensive pasture systems [57-62]. Other strategies to improve calving intervals involve preventing suckling for a period of about two weeks when calves are between two and three months-old, until ovulation is resumed. This may be achieved by temporary separation of the calf-dam pair [63,64] or by preventing suckling with nose-flaps [57]. The effectiveness of these practices on the reproductive performance of the herd is influenced by factors such as parity of the cows [65] or their body condition [63], and therefore several combinations of practices involving restricting lactation are being tested and studied.

Because weaning and cow-calf temporary separation have been proven useful to improve the reproductive efficiency of beef cattle herds raised on pasture systems [57,65], they are likely to be increasingly adopted, especially considering societal pressure to develop sustainable livestock production systems [12,61,66]. In this context, the influence of such practices on the wellbeing of calves needs to be carefully considered.

### Main stressors associated with weaning

At weaning beef calves are usually submitted to the sudden and simultaneous loss of the social contact with the dam and the milk she provided. The former involves the loss of access to the udder and thus suckling behaviour, and a break of the bond with the mother. Usually, weaning also involves changes in social and physical environment. Although these stressors can be presented separately, what normally happens is a superimposition of several or even all of them. Thus, it is difficult to discuss the relative importance of the loss of milk, the loss of the udder and the separation from its dam for the beef calf.

The motivation to maintain the social bond by both parties goes beyond obtaining milk, because besides nutrition nursing also provides emotional comfort to the young [67]. Nonetheless, six-month-old beef calves that were prevented from nursing with the use of nose-flaps displayed increased vocalization, walking and reduced playing, ruminating and grazing, indicating that cessation of nursing may contribute to the weaning distress response in beef calves even at this age [68]. Milk is a food rich in protein and energy, and in the case of beef cattle it has been estimated that the amount of milk produced by cows six to seven months after birth can provide approximately 30% of the metabolizable energy required for European breeds of calves raised on pasture [69-71]. Some studies reported a decrease in growth rate and even weight loss in beef calves weaned at around six months [6,59,72], whereas in others no change in growth was found [7,73]. Weaning distress appears to be greater in calves that suckle cows with higher milk yield and are heavier at weaning [74], but not when live weight is controlled [68]. Thus, differences are likely related to the development of the calf, and the amount and quality of solid food available before and after weaning in different studies.

In pasture systems it is common to move weaned calves to new paddocks, changing the physical environment, and possibly influencing the response to weaning. Though we are not aware of studies in beef cattle, it has been shown in studies with deer calves (*Cervus elaphus*) [75], foals [76] and piglets [77] that remaining in the same place after weaning reduces the effects of weaning stress. In confinement systems, animals are also usually subjected to new housing and diet, for example, changes from pasture or hay to concentrate feed [78]. Similarly in pasture-based systems it is quite common to isolate the calves during weaning for about a day in a corral, and then move them to a new paddock. In most cases, the corral or new paddock may be environments totally unknown to the calves; thus, they do not know the location of resources such as food, shade, or water source. Moreover, changing the physical environment can interfere with the animals' ability to recognize members of their group [79], which can generate social stress.

Although mixing of unfamiliar animals is less common when weaning beef cattle than other species, the mere fragmentation of stable groups during weaning can act as a stressor [80]. For example, the separation of a group of cows and their calves from the main herd five days before the day of weaning was sufficient to increase the concentration of cortisol in the blood of cortisol in the blood of the calves [81]. Further studies should consider the effect of social disruption on the overall response of beef cattle to weaning. This may be done comparing the response to weaning in groups that are submitted to social disruption and other that remain in their social group.

### Physiological and behavioural responses to weaning in beef calves

Some studies have addressed the physiological responses to weaning in calves. Abrupt weaning at six months causes increases in plasma cortisol [82] and norepinephrine [81]. Also, another study reported an increase in peripheral catecholamine concentration in response to separation, and a subsequent decrease when the same cows and calves were reunited [2]. An increase in plasma cortisol and heart rate also occurred after separation of dairy calves from their foster dams at three months of age [83]. Acute phase protein concentration also increases after weaning [84]. An increase in the ratio of neutrophils and lymphocytes [81], and a reduction in antioxidant enzyme activity of leukocytes [73], which indicate the presence of oxidative stress, has been described in beef calves weaned at seven months. A transitory reduction in immune function, peaking between d2 and d7 after weaning, was reported in grazed beef calves abruptly weaned at seven months [8].

Among the many behavioural changes taken as indicators of weaning stress, perhaps the most characteristic is the high frequency of vocalizations emitted by the calf [9,10,43]. Vocalizations by the young are thought to evoke maternal care [10,85] and the need to reunite with the dam [9]. According to evolutionary theories [86,87] a honest signal must fulfil four requirements: 1) there must be a degree of relatedness between sender and receiver of the signal; 2) the emission intensity and the benefit obtained should be proportional to the need for resources by the offspring; 3) emitting the signal has a fitness cost; and, 4) the receiver (the dam) must obtain a fitness benefit by providing resources to the signaller (the offspring). Thus, the vocalizations that typically follow weaning are considered a reliable signal of the emotional and physiological condition of the calf because the energy cost and high risk of attracting predators can be compensated by the high-value resources provided by the dam [13,85]. Moreover the high frequency vocalizations that are often associated with abrupt weaning may also indicate the animal's state of frustration for being unable to receive food, care or reunion with the mother [7,88].

An increase in general activity and walking frequency [6,82,83,89], and pacing [1,5,7,74,89,90] have been reported in beef calves immediately after weaning. In general, weaned calves increase time they remain standing, and remain little time resting [5,6,83] compared with pre-weaning time budgets. These behaviours, together with vocalizations, have also been interpreted as a sign of motivation to reunite with the dam [88]. Some changes in the feeding behaviour of calves can also be observed immediately after weaning. Usually there is a reduction in the time grazing and consuming other solid foods [5,6,68,74,83], which is accompanied by a reduction in rumination time, probably due to a change in diet intake and composition [1,83].

Weaning also triggers a marked and progressive change in the general behaviour repertoire of calves, which starts to adopt a behaviour pattern more typical of adult cattle, characterized by the predominance of maintenance activities [18]. Whereas during early life most social interactions of calves involve the dam, after separation the relationships with the other animals in the group become more important. After weaning playing frequency is reduced [7,91,92] and aggressive and affiliative interactions within groups of calves increase [6,43,91]. Also, the behaviours within groups of weaned calves becomes more synchronized [5,43], with greater spatial cohesion and social interaction than among suckling calves [43]. This change probably occurs because before weaning the behaviour of the calf is more associated to that of its dam.

### Strategies used to reduce weaning distress

Different weaning methods have been used with the goal of reducing the negative consequences of weaning on behaviour, performance and welfare. Some of these methods aim to enable the calf to cope with the change in diet that accompanies the separation from the dam, while others attempt to mimic the natural weaning process, by causing the loss of milk to occur before the final separation from the dam.

Previous contact with the solid food that will be provided after weaning may result in a gradual replacement of milk with solid food while the animal is still in contact with the mother, thus encouraging independence from the mother as early as possible [10]. Practices such as "creep feeding" or "creep grazing" in which the calf has access to feed or pasture of high quality, respectively, have been successfully used to stimulate the calf to eat solid food and thus progressively reduce its nutritional and social dependence on the cow [58,93,94]. Although especially important in the case of early weaning, these techniques should also be encouraged when weaning is carried out at conventional ages, as it also produces positive results [95]. Beef calves conditioned to hay prior to weaning ate longer and had a reduced behavioural distress response at weaning compared to calves that had not had prior contact with hay [5]. Since feeding behaviour is influenced by social facilitation and learning from peers, the inclusion in the group of animals that are familiar with the food may encourage consumption of the novel food by weanling calves, thus improving their welfare. However, although there are anecdotal reports of positive results, the few studies that evaluated the method found no benefit to the health or growth of these animals, and suggested the possibility that the presence of unfamiliar animals may cause stress [96,97].

The transition to a fully solid diet while the calf is still with the dam has been forced by the use of nose plates in calves, which prevent nursing but allow the consumption of solid food [1,6,7,73]. Six to seven-month old beef calves that were weaned using nose-flaps over 5 or 14 days vocalized and walked less, had fewer agonistic interactions and spent more time eating and lying down, than calves separated abruptly from their dams when finally weaned [6]. Although the use of nose-flaps reduced some of the behaviours associated with stress at weaning, in some cases [6,73] these animals also had lower average daily weight gain. Moreover, the welfare of calves may also be impoverished by the fact that after the placement of nose-flaps there are several attempts to nurse and the calf stays in closer proximity to the cow [7,68], which suggests that these calves may be frustrated by not having access to a resource that is otherwise apparently available.

Another weaning method involves separating the cow-calf pair through a fence for a few days before the definitive separation, which allows partial physical contact, while preventing suckling [5,7,73,89,91]. However, results from studies investigating this method of weaning are contradictory, possibly due to different duration of the separation, timing of the observations, added to genetic and environmental variation between the studies. In one study, calves that had been separated from their mothers through fences during a short period of time before the final separation had higher daily weight gain, spent less time walking, emitted fewer vocalizations, and spent more time eating and lying than abruptly weaned calves [5]. In another study, beef calves had a higher frequency of behaviours indicative of stress during temporary separation from the mother through a fence, than calves that were separated but had no contact with their mothers [89]. No benefits for weight gain or biomarkers of oxidative stress were found when calves were weaned after seven days of fenceline separation [73]. Calves weaned after 14 days of fenceline separation grew less than those weaned abruptly, and vocalized more frequently and over a greater number of days during the period of partial separation [7]. Furthermore, in two of these studies [5,7], during the first days of fenceline separation calves spent more than half the time near the fence that separated them from the cows, suggesting a high motivation to reunite with the dam. A similar response was reported in fence-separated foals [91] and lambs [98]. Foals stayed mostly within 4 m of the fenceline during the first 5 hours after separation from the dam by a fence [91]. Also, lambs submitted to a similar weaning process were observed inside a 1 m strip from the fenceline at least two times per hour in the second and third day after fenceline separation [98]. Thus, it is possible that the fact of being unable to fulfil a strong motivation to suckle or make physical contact with the dam may be a source of stress and frustration for these young animals.

## Summary and Conclusions

Greater knowledge of the physiological mechanisms involved in the natural weaning compared with artificial disruption of the maternal-young bond may bring some light into the underlying mechanisms. Also, clearer understanding of the relationship between physiological changes and the resulting behaviours associated with weaning are needed, in order to assess the magnitude of the weaning distress response. This may also help guide the development of effective practices to minimize the stress associated with weaning. Most information in this area comes from studies with rodents, which may not translate well to ungulates and specifically domestic cattle. Thus, understanding the physiological changes during natural, early abrupt weaning and weaning with the alternative methods covered earlier in this review may help propose hypotheses regarding the relative contribution of the social and nutritional losses to the calf.

Although weaning is considered a major source of stress for beef calves, it is also considered a necessary practice to ensure reproductive efficiency, accelerating rebreeding of the dam postpartum, and thus increasing pregnancy rates. Therefore, as weaning practices will probably be widely applied in grazing systems, techniques to minimize weaning distress should be investigated and developed, and included in practical management. Methods currently applied in attempt to reduce distress associated with weaning involve mimicking the gradual changes in diet and social bond of natural weaning. Studies assessing such methods have provided conflicting results, with some suggesting that step weaning using fenceline separation or nose-flaps may be beneficial, others concluding that they do not influence the outcome for the calves or that it may even impoverish welfare to some extent. Instead of reducing the magnitude of the stress caused by abrupt weaning, these practices may redistribute the response in two episodes, one when the motivation to suckle or establish full physical contact are prevented, and another one at the moment of the definitive separation. These results lead to further questions about whether these methods actually provide an overall benefit for the calves and justify the extra management involved, such as moving animals for nose-flap fitting and verification of its permanence, or preparation of appropriate fencing to keep cows and calves apart.

Further studies should assess possible influences of milk yield, pregnancy and metabolic state of the cow during lactation, as well as the influence of food availability and quality, both before and after weaning, on the effect of these methods on the calves' growth, behaviour and physiology. Another unexplored issue is the possible influence of previous contact with humans and habituation to handling, on the response to weaning. Weaning stress may be reduced, for example, by exposing the calves to the new environment features they will find after weaning, such as feedstuffs, location of drinking water, new social partners, humans and handling devices. Furthermore, as younger weaning ages are being increasingly recommended and adopted in pasture systems, in younger animals the sudden shift to a fully solid diet may have greater negative impact than for calves weaned at conventional ages. Thus, the influence of age at weaning on the distress response, as well as the effectiveness of current alternative weaning methods for reducing weaning distress in younger animals need to be assessed. Studies should focus especially at the nutritional aspects associated to weaning, as these are likely to be more important at younger ages.

## Competing interests

The authors declare that they have no competing interests.

## Authors' contributions

DEH drafted the manuscript and compiled the literature. All authors made substantial inputs to the review, critically discussed the progressing manuscript and approved the final manuscript.
